# Anifrolumab for Adolescent Discoid Lupus Erythematosus

**DOI:** 10.1001/jamanetworkopen.2023.38200

**Published:** 2023-10-18

**Authors:** Katharina S. Shaw, Ahmad Rajeh, Todd Le, Philip J. Kahn, Vikash S. Oza, Lisa M. Arkin, Ruth Ann Vleugels

**Affiliations:** 1 Department of Dermatology, Perelman School of Medicine, University of Pennsylvania, Philadelphia; 2 Section of Dermatology, Children’s Hospital of Philadelphia, Philadelphia, Pennsylvania; 3 Department of Dermatology, Brigham and Women’s Hospital, Harvard Medical School, Boston, Massachusetts; 4 Department of Dermatology, University of Wisconsin School of Medicine and Public Health, Madison; 5 Department of Pediatrics, University of Wisconsin School of Medicine and Public Health, Madison; 6 Division of Pediatric Rheumatology, Department of Pediatrics, Hassenfeld Children’s Hospital at NYU Langone Health, New York University Grossman School of Medicine, New York; 7 The Ronald O. Perelman Department of Dermatology, New York University Grossman School of Medicine, New York; 8 Dermatology Program, Division of Immunology, Boston Children’s Hospital, Boston, Massachusetts

## Abstract

This case series describes the outcomes among adolescent patients with systemic lupus erythematosus and refractory discoid lupus erythematosus treated with anifrolumab.

## Introduction

Anifrolumab, a human monoclonal antibody targeting type I interferon receptor, was approved in 2021 for adults with systemic lupus erythematosus (SLE) and has since emerged as an efficacious agent for refractory discoid lupus erythematosus (DLE).^1^ Rapid clinical improvement has been attributed to anifrolumab’s selective antagonism of type I interferon signaling, a known factor associated with DLE disease activity.^2^ However, its utility for adolescents with SLE and recalcitrant DLE remains unknown.

## Methods

This multicenter retrospective case series used medical records of adolescent patients treated with anifrolumab at Boston Children’s Hospital, Hassenfeld Children’s Hospital, and University of Wisconsin Hospital. The study was deemed exempt by each participating site’s institutional review board, and consent was waived as data were deidentified. Inclusion criteria were adolescent patients with SLE and recalcitrant DLE seen between August 1, 2022, and June 30, 2023, who received 1 or more dose of anifrolumab. Electronic health records were reviewed for demographics, clinical features, treatment data, and adverse events. This study followed the reporting guideline for case series. Analyses were performed using R, version 4.2.2 (R Project for Statistical Computing). *P* values were calculated using the Mann-Whitney test, and 2-sided *P* <.05 was considered significant.

## Results

Seven adolescent patients with SLE (6 females; median age, 17 years [range, 14-20 years]) treated with anifrolumab (300 mg administered intravenously every 4 weeks; median, 6 doses [range 3-8 doses]) were identified (Table), including 1 patient whose early treatment response was previously published.^1^ Race, ethnicity, and/or ancestry were classified by parent or self-reporting. Four patients (57.1%) were Asian, 2 (28.6%) were Black or African American, and 1 (14.3%) was White. All had DLE recalcitrant to standard therapies at time of anifrolumab initiation (median number of prior systemic treatments, 4 [range, 3-11]). Primary outcome was reduction in Cutaneous Lupus Erythematosus Disease Area and Severity Index score (CLASI Activity [CLASI-A] score, 0-70; CLASI Damage [CLASI-D] score, 0-56). SLE activity (SLE Disease Activity Index 2000 [SLEDAI-2K] score) was also assessed. All patients demonstrated substantial improvement in cutaneous disease activity after initiating anifrolumab (Figure, A-E). Mean (SE) decrease and mean (SE) percentage decrease in CLASI-A scores were 18.0 (8.9) and 72.1% (9.4%), respectively, after 1 month (*P* =.01), which was sustained at 6-month follow-up. In addition, mean (SE) decrease in SLEDAI-2K score at last follow-up was 7.0 (6.2), reflecting overall improvement in SLE disease activity. No significant change in CLASI-D score, which quantifies scarring and dyspigmentation from antecedent DLE disease activity, was observed (Figure, F). One patient (14.3%) experienced recurrent herpes simplex virus type 1 (HSV-1) reactivation (Table).

**Table.** Patient Characteristics and Treatment Response to Anifrolumab

Patient No.	Sex^a^	Prior therapies^b^	Therapies concomitant with ANI	Therapies discontinued or tapered after ANI initiation	Baseline CLASI-A score	CLASI-A score at last follow-up (mo)	CLASI-A score change (% change)	Baseline CLASI-D score	CLASI-D score at last follow-up (mo)	CLASI-D score change (% change)	Baseline SLEDAI-2K score	SLEDAI-2K score at last follow-up (mo)	SLEDAI-2K score change (% change)	Adverse events	Extracutaneous SLE disease activity requiring additional therapy
1	F	HCQ, MTX, MMF, SCS	HCQ, MMF	SCS	27	0 (6)	27 (100)	17	17 (6)	0	24	4 (6)	20 (83.3)	Recurrent HSV-1 reactivation	None
2	F	HCQ, MMF, SCS	HCQ, MMF, SCS	SCS	25	1 (9)	24 (96.0)	10	10 (9)	0	9	2 (9)	7 (77.8)	None	Pericarditis
3	F	HCQ, MMF, ILS, SCS	HCQ, MMF	MMF	7	2 (3)	5 (71.4)	5	5 (3)	0	4	0 (3)	4 (100)	None	None
4	F	HCQ, quinacrine, MMF, dapsone, IVIG, RTX, SCS	HCQ, MMF, SCS	None	27	3 (6)	24 (88.9)	11	11 (6)	0	22	10 (6)	12 (54.5)	None	Proteinuria
5	M	HCQ, MTX, MMF, AZA, belimumab, SCS	HCQ, MMF, SCS	SCS	11	1 (6)	10 (90.9)	5	3 (6)	2 (40.0)	6	2 (6)	4 (66.7)	None	None
6	F	HCQ, MTX, MMF, AZA, leflunomide, thalidomide, cyclophosphamide, RTX, belimumab, IVIG, SCS	HCQ, MMF, IVIG	IVIG	36	1 (9)	35 (97.2)	22	20 (9)	2 (9.1)	12	8 (9)	4 (33.3)	None	Organic brain syndrome
7	F	HCQ, MTX, MMF, SCS	HCQ, MMF, SCS	SCS	20	0 (3)	20 (100)	4	4 (3)	0	18	4 (3)	14 (77.8)	None	None

Abbreviations: ANI, anifrolumab; AZA, azathioprine; CLASI-A, Cutaneous Lupus Disease Area and Severity Index Activity (scores 0-70, where higher scores indicate more severe cutaneous disease activity); CLASI-D, Cutaneous Lupus Disease Area and Severity Index Damage (scores 0-56, where higher scores indicate more severe cutaneous damage); HCQ, hydroxychloroquine; HSV-1, herpes simplex virus type 1; ILS, intralesional corticosteroids; IVIG, intravenous immunoglobulin; MMF, mycophenolate mofetil; MTX, methotrexate; RTX, rituximab; SCS, systemic corticosteroids; SLE, systemic lupus erythematosus; SLEDAI-2K, Systemic Lupus Erythematosus Disease Activity Index 2000 (range, 0-105, where higher scores indicate greater SLE activity).

^a^ This cohort included Asian, Black or African American, and White patients between 14 and 20 years of age. Race and ethnicity were classified by the parent or by self-reporting in the electronic medical record.

^b^ Prior therapies were considered a failure if undertaken for 12 weeks without adequate disease control or not tolerated by the patient for any reason.

**Figure.**
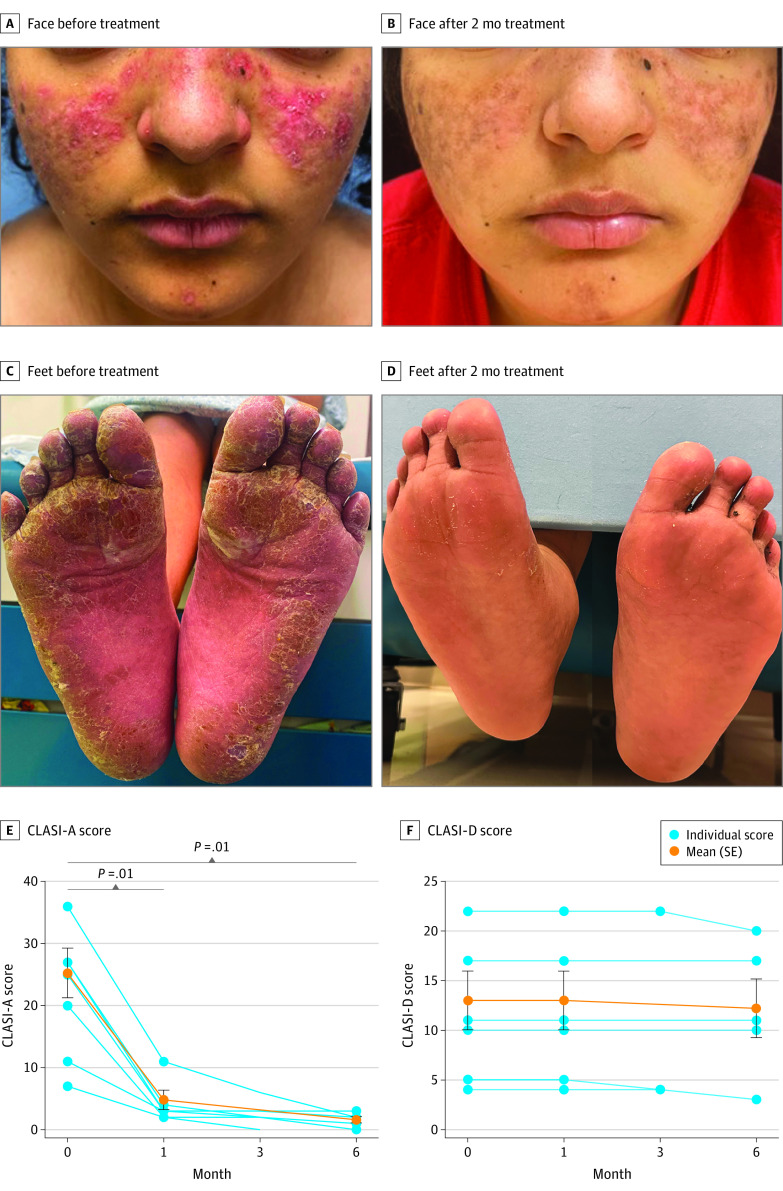
Clinical Improvement of Recalcitrant Discoid Lupus Erythematosus in Adolescent Patients With Systemic Lupus Erythematosus Treated With Anifrolumab Representative photographs of 2 adolescent patients demonstrate improvement in discoid lesions on the face (A) and feet (C) after 2 months of treatment (B and D, respectively) with anifrolumab. Line graphs showing Cutaneous Lupus Erythematosus Disease Area and Severity Index (CLASI) Activity (CLASI-A [scores 0-70, where higher scores indicate more severe cutaneous disease activity]) (E) and Damage (CLASI-D [scores 0-56, where higher scores indicate more severe cutaneous damage]) (F) scores for 7 patients before treatment and at month 1, 3, and/or 6 of anifrolumab treatment. Blue circles represent individuals. The orange line indicates mean (SE) CLASI-A (E) and CLASI-D (F) scores calculated before treatment and at months 1 and 6. Patients with missing values at month 6 (n = 2) were excluded from mean calculation. Error bars indicate SEs.

## Discussion

DLE is characterized by irreversible scarring and disfigurement in cosmetically sensitive areas. Rapid and effective treatment is critical to minimize long-term cosmetic sequelae that may affect self-esteem and psychosocial functioning, particularly among adolescent patients.^3^

Anifrolumab recently emerged as a promising therapeutic option for adult patients with refractory DLE, as demonstrated in several case series and a retrospective cohort study from our group.^1,2^ We report first outcomes data of adolescent patients with SLE and refractory DLE treated with anifrolumab. Not only did we observe significant improvement in skin disease with just 1 dose of anifrolumab (including mean 18-point reduction in CLASI-A score, when a reduction by 3-4 points is considered clinically meaningful), but clinical improvement occurred within a matter of weeks. Infusions were well tolerated. We did observe 1 case of recurrent HSV-1 reactivation, which is consistent with increased risk for viral infections reported in original phase 3 trials among adult patients with SLE.^4^ Additionally, while all patients demonstrated improvement in overall SLE disease activity, 3 patients experienced SLE manifestations (pericarditis, worsening proteinuria, or progressive organic brain syndrome) requiring additional therapy (Table). All 3 patients had a history of severe SLE not in remission at time of anifrolumab initiation, and 2 patients reported nonadherence to adjunctive mycophenolate mofetil prior to worsening of kidney disease and neurologic involvement, respectively.

Our findings suggest that anifrolumab is associated with rapid and sustained improvement of recalcitrant DLE among adolescent patients with SLE. Although this study is limited by its small sample size and retrospective nature, it is the first series, to our knowledge, to describe skin disease response to anifrolumab in this population.

## Notes

Supplement.
Data Sharing Statement
