# Supplementary material for: Anifrolumab for Adolescent Discoid Lupus Erythematosus
Source: JAMA Netw Open. 2023 Oct 18;6(10):e2338200. doi: 10.1001/jamanetworkopen.2023.38200 (PMC10585408; doi:10.1001/jamanetworkopen.2023.38200)
Supplement: Supplement. — Data Sharing Statement [file jamanetwopen-e2338200-s001.pdf]

Data Sharing Statement

Shaw. Anifrolumab for Adolescent Discoid Lupus Erythematosus. *JAMA Netw Open*. Published October 18, 2023. doi:10.1001/jamanetworkopen.2023.38200

Data
Data available: No
